# The AIM2 inflammasome is activated in astrocytes during the late phase of EAE

**DOI:** 10.1172/jci.insight.155563

**Published:** 2022-04-22

**Authors:** William E. Barclay, Nupur Aggarwal, M. Elizabeth Deerhake, Makoto Inoue, Toshiaki Nonaka, Kengo Nozaki, Nathan A. Luzum, Edward A. Miao, Mari L. Shinohara

**Affiliations:** 1 Department of Immunology, Duke University School of Medicine, Durham, North Carolina, USA.; 2 Department of Comparative Biosciences, College of Veterinary Medicine, University of Illinois at Urbana-Champaign, Urbana, Illinois, USA.; 3 Department of Molecular Genetics and Microbiology, Duke University School of Medicine, Durham, North Carolina, USA.

**Keywords:** Autoimmunity, Neuroscience, Autoimmune diseases, Innate immunity, Multiple sclerosis

## Abstract

Inflammasomes are a class of innate immune signaling platforms that activate in response to an array of cellular damage and pathogens. Inflammasomes promote inflammation under many circumstances to enhance immunity against pathogens and inflammatory responses through their effector cytokines, IL-1β and IL-18. Multiple sclerosis and its animal model, experimental autoimmune encephalomyelitis (EAE), are autoimmune conditions influenced by inflammasomes. Despite work investigating inflammasomes during EAE, little remains known concerning the role of inflammasomes in the central nervous system (CNS) during the disease. Here, we used multiple genetically modified mouse models to monitor activated inflammasomes in situ based on oligomerization of apoptosis-associated speck-like protein containing a CARD (ASC) in the spinal cord. Using inflammasome reporter mice, we found heightened inflammasome activation in astrocytes after the disease peak. In contrast, microglia and CNS-infiltrated myeloid cells had few activated inflammasomes in the CNS during EAE. Astrocyte inflammasome activation during EAE was dependent on absent in melanoma 2 (AIM2), but low IL-1β release and no significant signs of cell death were found. Thus, the AIM2 inflammasome activation in astrocytes may have a distinct role from traditional inflammasome-mediated inflammation.

## Graphical abstract

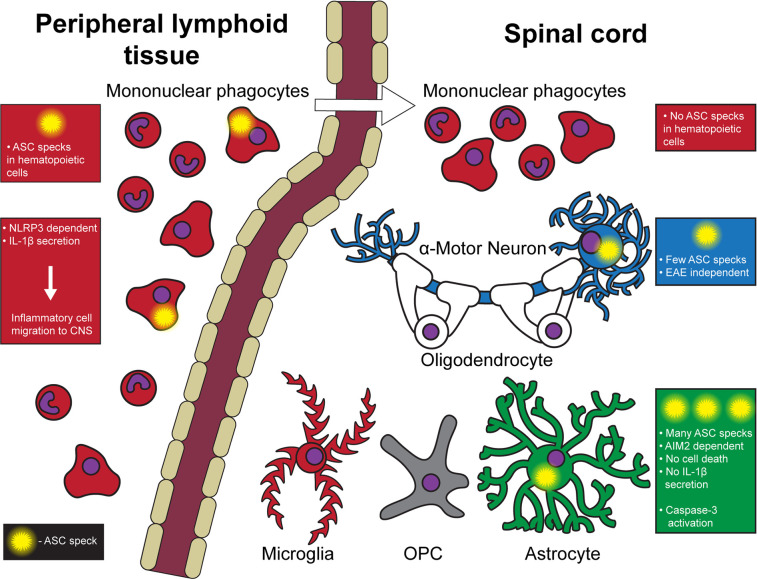

## Introduction

Multiple sclerosis (MS) and its mouse model, experimental autoimmune encephalomyelitis (EAE), are demyelinating neurodegenerative diseases punctuated by inflammatory immune reactions in the central nervous system (CNS). Inflammasomes are sensors of a wide range of microbe-associated molecular patterns and damage-associated molecular patterns and induce inflammation (1). In EAE, the NLRP3 inflammasome has a particularly well-documented role in the peripheral immune system in promoting immune cell recruitment to the CNS through the generation of the inflammatory cytokines IL-1β and IL-18 by peripheral myeloid cells (2–6). However, despite much work centered on inflammasomes in the peripheral immune response, the role of inflammasomes in the CNS is far less understood.

Inflammasomes are distinct from other pattern recognition receptors in mode of signaling and downstream effector function. First, a sensor (e.g., NLRP3, absent in melanoma 2 [AIM2]) forms a scaffold, to which the inflammasome adaptor apoptosis-associated speck-like protein containing a CARD (ASC) binds and polymerizes (called the “ASC speck”). Pro–caspase-1 associates with the polymer, then self-cleaves to become proteolytically active caspase-1, which further activates downstream substrates, including pro–IL-1β, pro–IL-18, and the pore-forming protein, gasdermin-D (GSDMD). Cleaved GSDMD forms a pore in cellular membranes and induces pyroptosis, resulting in a release of mature IL-1β and IL-18 to the extracellular space. In MS, genetic variation in inflammasome signaling pathways was reported (7, 8). Particularly, the NLRP3 inflammasome was demonstrated to be a prognostic factor and a therapeutic target in primary progressive MS (9). In EAE, NLRP3 and ASC are necessary for passive and standard (Type-A) active EAE (2–4). NLRP3 inflammasome activation in macrophages and dendritic cells in secondary lymphoid organs results in IL-1β and IL-18 release, which induces expression of chemokines and their receptors required for leukocyte CNS entry (2). The NLRP3 inflammasome is activated by several ligands, including extracellular ATP (1, 6). Reduction of extracellular ATP by CD39 on conventional DCs is associated with reduced NLRP3 inflammasome activation, which subsequently reduces proliferation and differentiation of pathogenic Th1 and Th17 cells during EAE (6). We have also demonstrated that the NLRP3 inflammasome can be dispensable for EAE induction if the innate immune system is strongly activated with aggressive immunization schemes (Type-B EAE) (10).

As inflammasome activation is a posttranslational process, the expression of inflammasome components does not necessarily indicate their activation. Thus, separately assessing both expression and activation of inflammasomes is critical. Recent studies indicated that microglia, astrocytes, and neurons can activate inflammasomes; most identification was performed ex vivo (9, 11–18). However, defining inflammasome activation in situ is critical because CNS-resident cells significantly alter their behavior once isolated from tissues. So far, only a limited number of studies have demonstrated unequivocal activation of inflammasomes in situ in the CNS (11–13).

In this report, we identified activated inflammasomes in the CNS during EAE using the ASC-Citrine mouse line, which allows in situ detection of activated inflammasomes (19). Our study identified maximal inflammasome activation in the spinal cord (SC) after the EAE peak, which contrasts with the inguinal lymph nodes (iLNs), in which inflammasome activation was present before symptomatic disease. Unexpectedly, neither microglia nor CNS-infiltrated myeloid cells were the primary cells with activated inflammasomes in SC during EAE. Instead, we detected inflammasome activation mainly in astrocytes and limited inflammasome activation in motor neurons. Furthermore, we found that the AIM2 inflammasome was activated in astrocytes during EAE. However, even with AIM2 inflammasome activation, astrocytes did not clearly undergo cell death and had poor *Il1b* gene expression, suggesting the possibility that AIM2 inflammasome activation in astrocytes serves a different purpose than traditional inflammasome-mediated inflammation.

## Results

### Inflammasome activation in the SC at a late stage of EAE.

**Figure 1:**
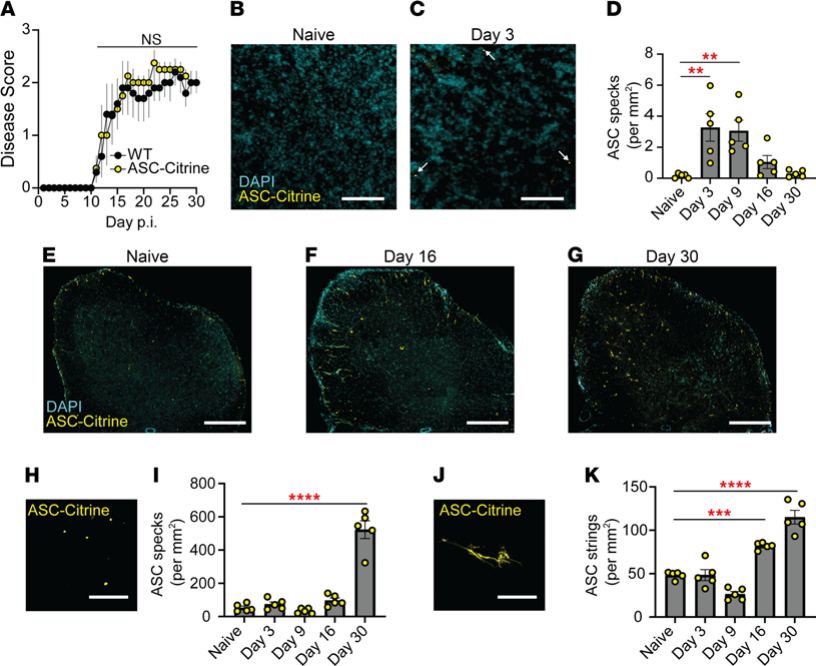
Inflammasome activation in the CNS during late EAE. (**A**) EAE disease score of WT (*n* = 5) versus ASC-Citrine mice (*n* = 4). Mann-Whitney *U* test of total AUC for disease was used. Each data point represents a mean value among a group. (**B**–**D**) Representative images (**B** and **C**) and quantification (**D**) of ASC specks in the iLNs of ASC-Citrine mice during EAE. Each data point represents an average value from 2 cross sections of both iLNs (25 μm thickness) per mouse. *n* = 5 mice. One-way ANOVA, *P* = 0.0006, with Dunnett’s multiple comparisons test. Scale bar is 20 μm. (**E**–**G**) Representative images of SC from ASC-Citrine mice at indicated time points during EAE. Scale bar is 300 μm. (**H**–**K**) Representative images (**H** and **J**) and quantification (**I** and **K**) of ASC specks (**H** and **I**) and ASC strings (**J** and **K**) in SC from ASC-Citrine mice during EAE. Scale bar is 10 μm. Each data point represents a value from 1 mouse (*n* = 5). Two coronal cross sections (25 μm thickness) of L5 SC from 1 mouse were quantified manually and averaged. One-way ANOVA with Dunnett’s multiple comparisons tests (**D**, **I**, and **K**). **P < 0.01, ***P < 0.001, ****P < 0.0001. Error bars denote mean ± SEM (**A**, **D**, **I**, and **K**).

Inflammasome signaling is critical to EAE development in the peripheral lymphoid organs (2–5, 10). Yet, the extent and spatiotemporal distribution of inflammasome activation in the CNS during EAE is largely unknown. Classically, detection of inflammasome activation is performed by identifying cleaved caspase-1 by Western blotting (WB), which cannot be applied in situ. Therefore, we used a different molecular signature of inflammasome activation — the oligomerization of ASC, microscopically observed as the “ASC speck.” In this study, we used inflammasome activation reporter mice, which express ASC fused to a fluorescent Citrine protein (ASC-Citrine) (19). The ASC-Citrine reporter allows in situ detection of active inflammasomes by visualization of ASC specks (13, 19) (Supplemental Figure 1A; supplemental material available online with this article; https://doi.org/10.1172/jci.insight.155563DS1), and the presence of the reporter did not alter the disease course of EAE (Figure 1A). The ASC-Citrine reporter was validated for use in tissue by immunostaining against ASC in live spleen slice cultures following NLRP3 inflammasome activation (Supplemental Figure 1A).

Before evaluating inflammasome activation in the CNS, we first visualized and quantified ASC specks in the iLNs as the primary site of immune reaction to EAE induction. ASC specks were detected at 3 days postinduction (dpi) of EAE (Figure 1, B–D), which is well before the disease onset, and continually detected until 9 dpi (Figure 1D). However, ASC specks were almost undetectable by the point of disease peak (16 dpi) and after (Figure 1D). The cervical lymph nodes have also been noted as a site of primary immune reaction in some models of EAE (20, 21), but few ASC specks were detected there throughout disease (Supplemental Figure 1B). The SC of ASC-Citrine mice exhibited a much higher number of ASC specks than the iLNs (Figure 1, E–I), with a significant increase in the number of ASC specks in the later phase of EAE at 30 dpi (Figure 1, G and I). Further, in addition to ASC specks, we also observed atypical fiber-like ASC-Citrine signals, which we termed “ASC strings,” unique to the CNS (Figure 1, J and K). While different in magnitude, both ASC specks and ASC strings appeared with the largest increases at the later phase of disease, around 30 dpi (Figure 1, I and K). Due to the abundance of ASC specks and strings at 30 dpi, all subsequent analyses in SC were at 30 dpi, unless otherwise stated. This quantification was also performed manually, and all subsequent ASC speck and string quantification was performed using the Imaris software.

We have previously shown a subtype of EAE (Type-B EAE) that does not require the NLRP3 inflammasome in the peripheral lymphoid organs to develop EAE (10). In the CNS, Type-A and Type-B EAE had comparable numbers of ASC specks (Supplemental Figure 1, C and D), suggesting the more consistent connection of EAE severity and active inflammasome in the CNS than in the periphery. In sum, these results suggest that inflammasomes are activated in the SC during both Type-A and Type-B EAE and that their activation is heightened after peak disease.

### Inflammasome activation in non-BM-derived cells in the SC.

**Figure 2:**
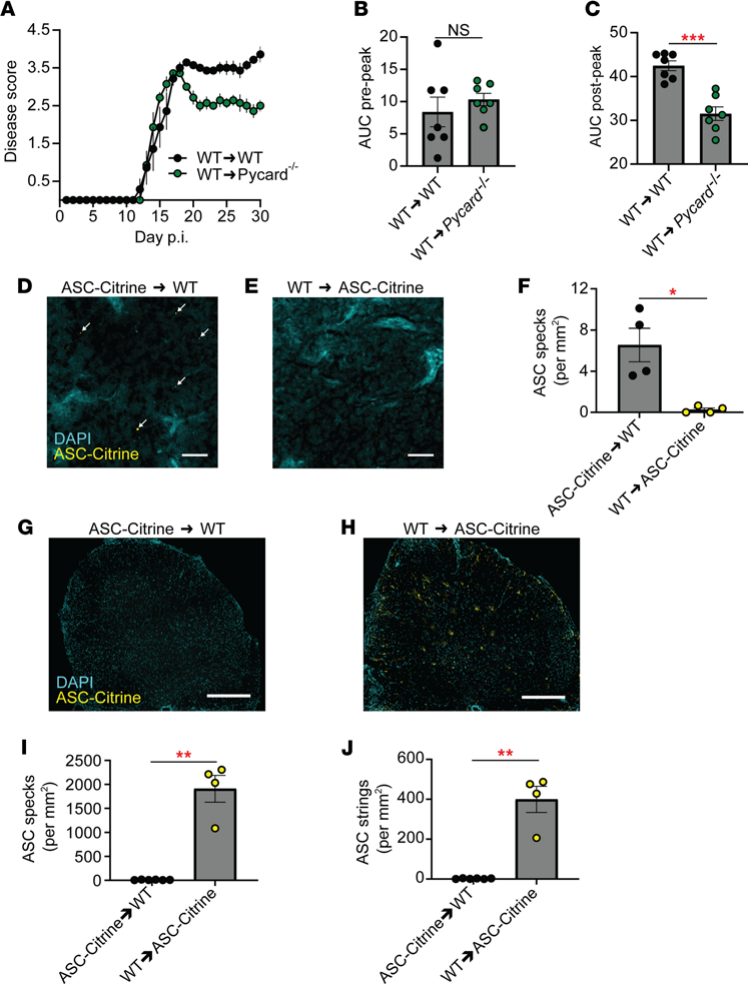
No inflammasome activation in hematopoietic cells in the CNS during EAE. (**A**) EAE disease scores of WT (BM donor)→WT (recipient) chimeras versus WT→*Pycard^–/–^* chimeras. *n* = 7, combined from multiple experiments. Each data point represents a mean value among a group. (**B** and **C**) Comparison of EAE disease severity of WT→WT chimeras versus WT→*Pycard^–/–^* chimeras. Each data point represents a value from 1 mouse (*n* = 7), combined from multiple experiments. AUC quantification of prepeak disease (**B**), AUC quantification of postpeak disease (**C**). (**D**–**F**) Representative images (**D** and **E**) and quantification (**F**) of ASC specks in the iLNs of ASC-Citrine→WT chimeras (**D**) versus WT→ASC-Citrine chimeras (**E**) at 3 dpi of EAE. Each data point represents a value from 1 mouse as an average of both iLNs (*n* = 4). Mann-Whitney *U* test was used. Scale bar is 20 μm. (**G**–**J**) Representative images (**G** and **H**) and quantification of ASC specks (**I**) and ASC strings (**J**) of SC from WT→ASC-Citrine BM chimeras (*n* = 4) versus ASC-Citrine→WT chimeras (*n* = 6) at 30 dpi of EAE. Each data point represents a value from 1 mouse. Mann-Whitney test was used (**B**, **C**, **F**, **I**, and **J**). Scale bar is 300 μm. *P < 0.05, **P < 0.01, ***P < 0.001. Error bars denote mean ± SEM (**A**–**C**, **F**, **I**, and **J**).

Because we identified inflammasome activation in the CNS during EAE, the role of ASC in nonhematopoietic cells was investigated with a BM chimera approach using CD45.1 congenic donor mice. We compared 2 groups of BM chimeras, generated by adoptively transferring WT BM cells to either WT or *Pycard*^–/–^ (ASC knockout) recipients (the extent of reconstitution is shown in Supplemental Figure 2A). Compared with WT recipients, *Pycard^–/–^* recipients demonstrated milder EAE after the disease peak (Figure 2, A–C), suggesting that ASC in nonhematopoietic cells affected EAE severity after the disease peak. Next, we sought to determine if CNS-infiltrated cells possess activated inflammasomes in the SC during EAE. Two groups of BM chimeras were compared; one group with ASC-Citrine BM donor cells to WT recipients and the other with WT BM cells to ASC-Citrine recipients. Reconstitution of approximately 90% of BM cells was confirmed (Supplemental Figure 2B), and no impact of ASC-Citrine expression on EAE development was confirmed (Supplemental Figure 2C). ASC specks were identified in the iLNs of WT recipients reconstituted with ASC-Citrine BM (Figure 2, D–F). However, unexpectedly, the mice showed no ASC specks or strings in the SC (Figure 2, G–J, and Supplemental Figure 2D). In contrast, ASC specks and strings were identified in the SC of ASC-Citrine recipients transferred with WT BM cells (Figure 2, G–J). This suggests that the source of inflammasome activation is CNS-resident cells in the SC.

### Inflammasome activation in CNS during EAE in astrocytes.

**Figure 3:**
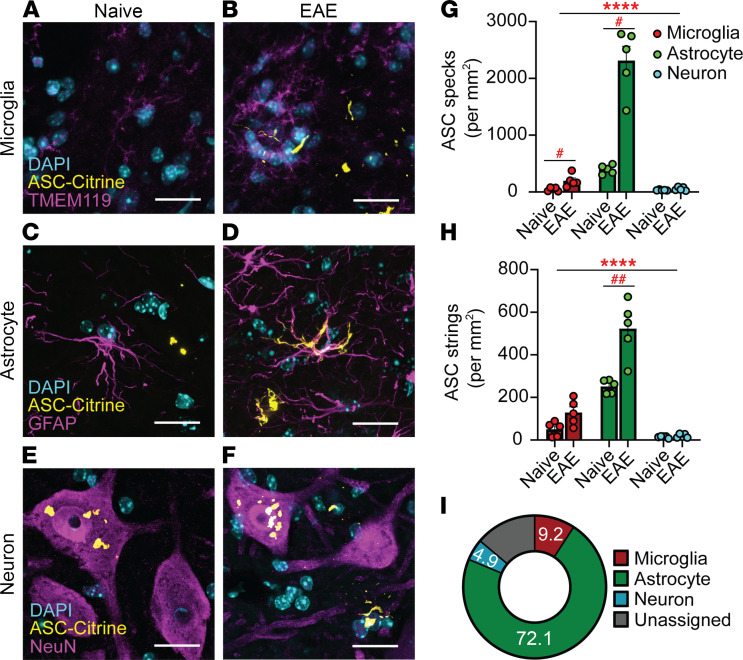
Identifying inflammasome activation in CNS cells with an immunofluorescence approach. (**A**–**F**) Representative images of ASC specks and strings counterstained with antibodies against TMEM119 for microglia (**A** and **B**), GFAP for astrocytes (**C** and **D**), and NeuN for neurons (**E** and **F**) in SC from naive (**A**, **C**, and **E**) versus 30 dpi EAE (**B**, **D**, and **F**) ASC-Citrine mice. Scale bar is 20 μm. (**G** and **H**) Quantification of ASC specks (**G**) and ASC strings (**H**) in microglia, astrocytes, and neurons in the ventral horn (VH) of SC from naive versus 30 dpi EAE ASC-Citrine mice. Each data point represents a value from 1 mouse (*n* = 5). Two-way repeated measures (RM) ANOVAs were used (main effect of cell type: ****P < 0.0001, **G** and **H**), with Holm-Šidák multiple comparisons test post hoc (^#^*P* < 0.05, ^##^*P* < 0.01). Error bars denote mean ± SEM. (**I**) Relative contribution of microglia, astrocytes, and neurons to total number of ASC specks in L5 SC at 30 dpi EAE. “Unassigned” indicates unidentified cell sources of ASC-Citrine signals.

We next sought to identify CNS-resident cells with activated inflammasomes during EAE. ASC specks and strings were identified, and then cell types were assigned by counterstaining to identify microglia (TMEM119), astrocytes (GFAP or ALDH1L1), neurons (NeuN), oligodendrocyte precursor cells (NG2), or mature oligodendrocytes (MBP) (Figure 3, A–F; and Supplemental Figure 2, E and F). The majority of ASC specks and strings were found in astrocytes, while a small number were classified as microglial and neuronal (Figure 3, G–I). Few ASC specks or strings were detected in oligodendrocyte precursor cells or mature oligodendrocytes (Supplemental Figure 2, G and H). In neurons, all ASC specks were found in cell bodies of choline acetyl transferase–positive (ChAT^+^) α motor neurons (ChAT^+^NeuN^+^) in the VH, but these neuronal ASC specks did not increase during EAE (Supplemental Figure 2I).

**Figure 4:**
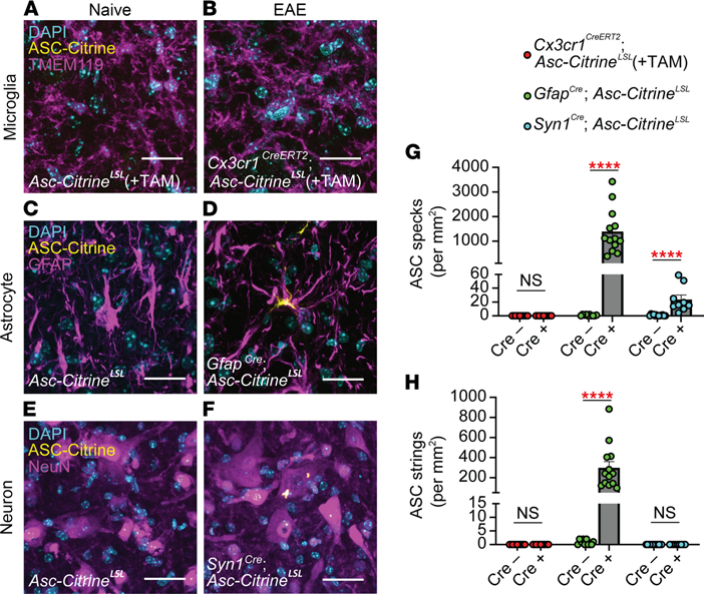
Identifying inflammasome activation in CNS cells by a mouse genetics approach. (**A**–**F**) Representative images of SC in *Asc-Citrine^LSL^* versus *Cx3cr1^CreERT2^* *Asc-Citrine^LSL^* mice (**A** and **B**), *Gfap^Cre/+^* *Asc-Citrine^LSL^* mice (**C** and **D**), and *Syn1^Cre/+^ Asc-Citrine^LSL^* mice (**E** and **F**) at 30 dpi of EAE. Mice for microglia evaluation were treated with tamoxifen. Scale bar is 20 μm. (**G** and **H**) Quantification of ASC specks (**G**) and ASC strings (**H**) in SC VH of mice indicated in **A**–**F**. Each data point represents a value from 1 mouse. Combined from multiple experiments. (**G** and **H**) Mann-Whitney *U* test was used. ****P < 0.0001. Error bars denote mean ± SEM.

We further validated these findings with a cell type–specific ASC-Citrine reporter approach by using *Asc*-*Citrine^LSL^* mice, which retain an LSL cassette upstream of the ASC-Citrine construct. To express ASC-Citrine in astrocytes and neurons in a cell type–specific manner, we used *Gfap^Cre^ Asc*-*Citrine^LSL^* and *Syn1^Cre^ Asc*-*Citrine^LSL^* mice, respectively. For microglia-specific ASC-Citrine expression, we used *Cx3cr1^CreERT2^ Asc*-*Citrine^LSL^* mice treated with tamoxifen (TAM) with a 6-week “washout” period to exclude ASC-Citrine expression in myeloid cells other than microglia (Supplemental Figure 3A) by taking advantage of the long half-life of microglia (gating strategy to evaluate microglia is shown in Supplemental Figure 3B). We considered that using the microglia-specific ASC-Citrine reporter mice was critical because the expression level of Tmem119, used in Figure 3, A, B, G, and H, decreases as EAE progresses (22) and may confound some image analysis. No alteration in EAE severity was confirmed in the group of mutant mice expressing Cre in targeted cell types (Supplemental Figure 3, C–E). The SC of these mutant mice was analyzed during EAE by confocal microscopy (Figure 4, A–F). Again, a high number of ASC specks and strings were confirmed in astrocytes by using the *Gfap^Cre^ Asc*-*Citrine^LSL^* mice, while few ASC specks or strings were identified in microglia (Figure 4, G and H). Neurons also showed a consistent but small number of ASC specks (Figure 4, G and H). Nonetheless, these data mirrored the results from antibody counterstaining in Figure 3, strongly indicating that inflammasome activation is predominantly in astrocytes during EAE.

### Limited induction of IL-1β–mediated inflammation by astrocytes during EAE.

**Figure 5:**
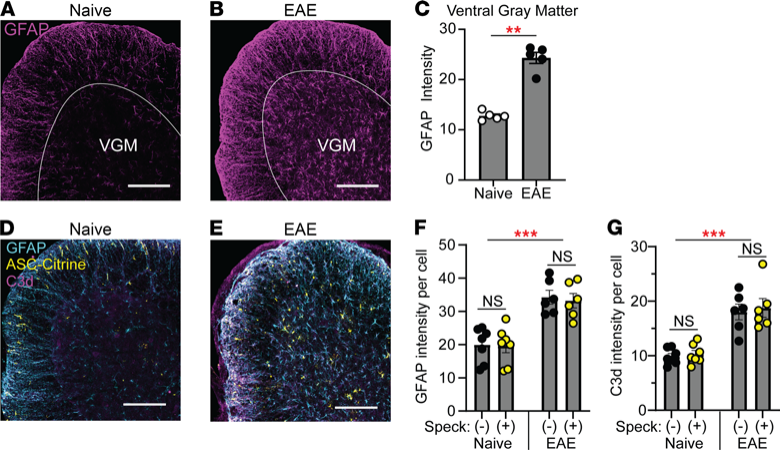
Evaluation of astrocytes with activated inflammasomes. (**A**–**C**) Representative images (**A** and **B**) and quantification (**C**) of total GFAP intensity in SC ventral gray matter from naive 30 dpi EAE mice. Scale bar is 200 μm. GFAP intensity was quantified as mean signal intensity of GFAP in the ventral gray matter (VGM) using ImageJ (NIH). Mann-Whitney *U* test was used (**C**). (**D**–**G**) Representative images (**D** and **E**) and quantification of GFAP intensity (**F**) and C3d intensity (**G**) per cell in gray matter SC astrocytes with and without ASC specks/strings from naive ASC-Citrine (*n* = 7) mice versus ASC-Citrine mice at 30 dpi of EAE (*n* = 6). Scale bar is 200 μm. Each data point represents a value from 1 mouse. Individual astrocytes were identified using the Imaris software, and the mean intensity per cell was quantified for GFAP and C3d. Two-way RM ANOVA was used with Holm-Šidák multiple comparisons test post hoc (**F** and **G**). **P < 0.01. ***P < 0.001. Error bars denote mean ± SEM (**C**, **F**, and **G**).

In EAE, astrocytes become activated in a process called astrogliosis; these activated cells gain a proinflammatory phenotype and are termed “reactive astrocytes” (23, 24). Here we investigated whether astrogliosis correlates with inflammasome activation. Astrogliosis was detected as increased GFAP intensity at 30 dpi (Figure 5, A–C), but GFAP intensity did not correlate on a per-cell basis with the presence of ASC specks (Figure 5, D–F). We similarly found no correlation between inflammasome activation and individual acquisition of the neurotoxic “A1” reactive astrocyte phenotype (25, 26), based on the A1 astrocyte marker C3d (27), though C3d expression was enhanced in astrocytes in aggregate during EAE (Figure 5, D, E, and G).

**Figure 6:**
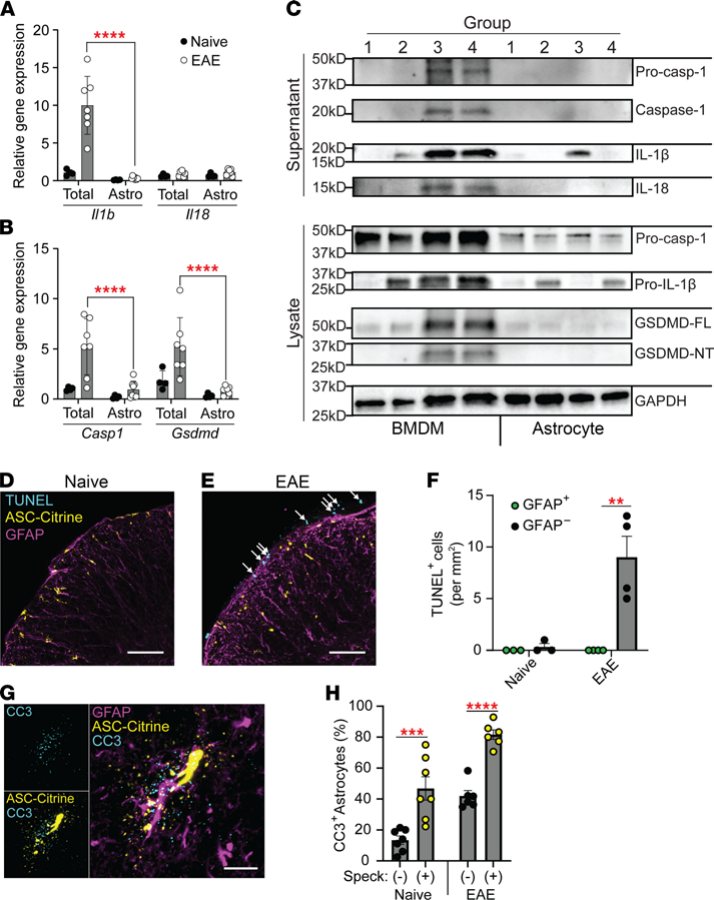
Outcomes of inflammasome activation in astrocytes. (**A** and **B**) RT-qPCR evaluation of gene expression for *Il1b* and *Il18* (**A**) and *Casp1* and *Gsdmd* (**B**) in SC total cells versus astrocytes isolated from naive mice (*n* = 4) and mice with EAE at 30 dpi (*n* = 7). Two-way RM ANOVAs were used with Holm-Šidák multiple comparisons test post hoc. Each data point represents a value from 1 mouse. (**C**) Representative images of Western blotting for inflammasome components in BMDMs versus primary cortical astrocytes. Cells indicated as group 1: unstimulated; group 2: treated with ultrapure LPS alone; group 3: pretreated with ultrapure LPS and stimulated with nigericin to activate the NLRP3 inflammasome; and group 4: pretreated with ultrapure LPS and stimulated with poly(dA:dT)/liposome to activate the AIM2 inflammasome. GSDMD-FL, uncleaved GSDMD; GSDMD-NT, cleaved N-terminal GSDMD. (**D**–**F**) Representative images (**D** and **E**) and quantification (**F**) of TUNEL staining of SC sections from naive (*n* = 3) and 30 dpi EAE ASC-Citrine mice (*n* = 4). Arrows indicate TUNEL^+^ cells (**E**). Two-way RM ANOVA was used (**F**). Scale bar is 75 μm. (**G** and **H**) Representative images (**G**) and quantification (**H**) of active caspase-3 (CC3) in SC astrocytes with and without ASC specks/strings. Evaluated from naive (*n* = *7*) and at 30 dpi EAE (*n* = 6) ASC-Citrine mice. Scale bar is 15 μm. Individual astrocytes were identified using the Imaris software and were quantified by CC3 puncta staining. Each data point represents a value from 1 mouse, combined from multiple experiments. Two-way RM ANOVA was used with Holm-Šidák multiple-comparison test post hoc. (**H**) **P < 0.01, ***P < 0.001, ****P < 0.0001. Error bars denote mean ± SEM (**A**, **B**, **F**, and **H**).

To evaluate astrocyte gene expression during EAE, we first reanalyzed publicly available data obtained with a *Gfap^Cre^*-driven RiboTag mouse system, which allows purification of astrocyte-specific RNA (National Center for Biotechnology Information’s Gene Expression Omnibus GSE100329) (28). Gene expression in total SC and SC astrocytes was compared between naive and 30 dpi EAE mice. We found low expression of *Il1b* and genes encoding inflammasome sensor proteins (Supplemental Figure 4A). Expression levels of *Il18* and *Casp1* were enriched in astrocytes independent of EAE (Supplemental Figure 4A). To validate these data, we evaluated gene expression by quantitative reverse transcription PCR (RT-qPCR) in total SC cells and astrocytes enriched by bead selection from naive and 30 dpi EAE mice. Astrocyte-enriched cells showed generally low expression of genes encoding proteins related to inflammasomes even during EAE. Notably, the expression of *Il1b*, *Casp1*, and *Gsdmd* was significantly lower in astrocyte-enriched cells than total SC cells (normalized to *Il1b* and *Casp1* expression in naive total SC cells for Figure 6, A and B, respectively). In the RT-qPCR analyses, a majority of genes shown in Supplemental Figure 4A had mRNA levels that were close to the detection limit, despite reasonably high total RNA amounts, suggesting the generally low expression of inflammasome-related genes in astrocytes. Under the low gene expression, we did not observe astrocyte enrichment of *Il18* and *Casp1* expression, as suggested in the RiboTag data (Supplemental Figure 4A). This was consistent with the limited detection of the inflammasome-related proteins caspase-1, IL-1β, and GSDMD in SC astrocytes of either naive mice or mice with EAE 30 dpi by immunostaining, despite robust detection in the spleen (Supplemental Figure 4, B–G). However, mild expression of GSDMD was detected in SC astrocytes following EAE induction (Supplemental Figure 4, D and G). Next, we performed WB to evaluate protein levels and inflammasome activation in vitro. We compared neonatal cortical astrocytes to BM-derived macrophages (BMDMs) as a positive control. We stimulated the NLRP3 or AIM2 inflammasome with nigericin or poly(dA:dT) liposomes, respectively, after ultrapure LPS pretreatment. Astrocytes released little caspase-1, IL-1β, and IL-18, excepting mild IL-1β release selectively in response to NLRP3 inflammasome stimulation (Figure 6C and Supplemental Figure 5, A and B). Notably, astrocytes produced less pro–caspase-1, pro–IL-1β, uncleaved GSDMD (GSDMD-FL), and cleaved N-terminal GSDMD (GSDMD-NT) compared with BMDMs, based on their presence in cell lysates (Figure 6C and Supplemental Figure 5, C–F). These results suggest that astrocytes have limited capacity to release IL-1β and IL-18 in response to AIM2 inflammasome stimulation. In contrast, NLRP3 inflammasome stimulation allows IL-1β release by astrocytes at least under this ex vivo condition.

Next, we investigated whether astrocytes with ASC specks or strings undergo cell death during EAE. We attempted to assess general cell death by TUNEL staining. TUNEL^+^ cells were present in the periphery of the SC at 30 dpi EAE, though no TUNEL^+^ astrocytes were detected (Figure 6, D–F), consistent with previous data demonstrating that astrocytes do not undergo significant cell death during EAE (29). Although normally not associated with canonical inflammasomes, we found enriched active caspase-3 in astrocytes with active inflammasomes in both ASC-Citrine (Figure 6, G and H) and *Gfap^Cre^ Asc-Citrine^LSL^* (Supplemental Figure 5G) mice, suggesting a possible connection between inflammasomes and caspase-3 activation in astrocytes. In summary, inflammasomes’ activation in astrocytes does not appear to lead to typical outcomes of inflammasome activation as seen in myeloid cells.

### AIM2 facilitates inflammasome activation in the CNS during EAE.

**Figure 7:**
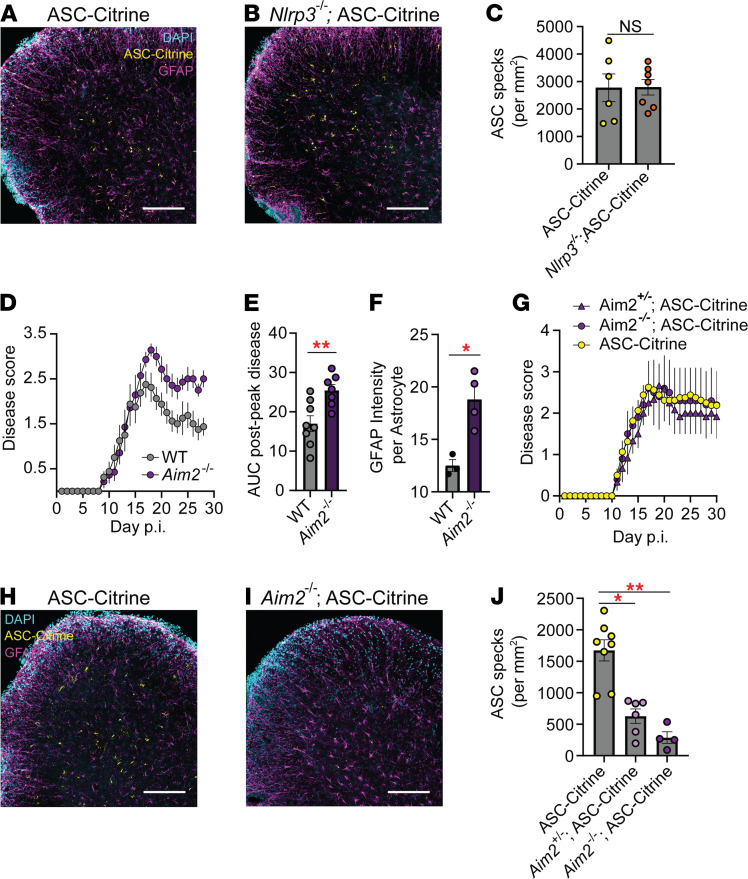
AIM2 inflammasome activation in astrocytes during EAE. (**A**–**C**) Comparison of ASC speck formation in indicated mouse groups. Representative images (**A** and **B**) and quantification of ASC specks (**C**) of SC sections from ASC-Citrine (*n* = 6) (**A**) versus *Nlrp3^–/–^* ASC-Citrine (*n* = 7) (**B**) mice at 30 dpi Type-B EAE (see Methods). Scale bar is 200 μm (**A** and **B**). Mann-Whitney *U* test was used (**C**). (**D** and **E**) EAE disease scores (**D**) and statistical evaluation of AUC (**E**) of WT (*n* = 8) versus *Aim2^–/–^* (*n* = 7) mice induced with Type-A EAE with low-dose *Mycobacterium tuberculosis* (*M*. *tuberculosis*) (50 μg/mouse). Mann-Whitney test of AUC was used to analyze postpeak disease (**D**). (**F**) Quantification of GFAP intensity per cell in gray matter SC astrocytes from WT (*n* = 3) versus *Aim2*^–/–^ (*n* = 4) mice at 30 dpi of Type-A EAE (see Methods) except for low-dose *M*. *tuberculosis* (50 μg/mouse). Unpaired 2-tailed *t* test was used. (**G**–**J**) Scoring Type-A EAE in ASC-Citrine (*n* = 8), *Aim2*^+/–^ ASC-Citrine (*n* = 6), and *Aim2*^–/–^ ASC-Citrine (*n* = 4) mice (**G**). Representative images (**H** and **I**) and quantification of ASC specks (**J**) of SC sections at 30 dpi EAE. Scale bar is 200 μm (**H** and **I**). One-way ANOVA was used (*P* < 0.0001) with Dunnett’s multiple comparisons test post hoc (**J**). Each data point represents a mean value among a group (**D** and **G**) or a value from 1 mouse (**C**, **E**, **F**, and **J**). *P < 0.05, **P < 0.01. Error bars denote mean ± SEM (**C**–**G** and **J**).

To further assess which inflammasome is activated in astrocytes during EAE, we selected NLRP3 and AIM2 among the inflammasome sensors based on their expression by astrocytes during EAE by the RiboTag raw transcript data (Supplemental Figure 4A). As *Nlrp3^–/–^* mice are resistant to standard EAE, we used the Type-B EAE model to induce EAE in the *Nlrp3^–/–^* background (3, 10) and confirmed that ASC-Citrine mice and *Nlrp3^–/–^* ASC-Citrine developed similar disease course and severity, as expected (Supplemental Figure 6A). Here, *Nlrp3*^–/–^ ASC-Citrine mice still showed comparable numbers of ASC specks with ASC-Citrine mice (Figure 7, A–C), suggesting that NLRP3 is dispensable in CNS inflammasome activation during EAE. Next, we tested the AIM2 inflammasome. Congruent with recent reports (14, 30), we found AIM2 to be protective in EAE, as demonstrated by more severe disease in *Aim2^–/–^* mice predominantly after disease peak, when induced with a low-dose adjuvant (50 μg *M. tuberculosis*/mouse) (Figure 7, D and E). The immune phenotype of *Aim2^–/–^* mice in SC, iLNs, and spleen at 16 dpi did not show statistically significant changes compared to WT mice, although a trend of increased T cells, microglia, and macrophages was observed, possibly reflecting the disease severity of *Aim2^–/–^* mice (Supplemental Figure 6, B–D), together with enhanced astrogliosis (Figure 7F and Supplemental Figure 6E).

Next, we investigated the role of the AIM2 in astrocyte inflammasome activation during EAE. To do so, we sought an EAE condition for *Aim2^–/–^* mice to develop comparable EAE severity. Despite increased EAE severity in *Aim2^–/–^* mice, an increased adjuvant dose (200 μg *M. tuberculosis* per mouse) elicited comparable EAE scores between WT and *Aim2^–/–^* mice (Figure 7G). Here, a strong gene dosage effect of AIM2 on ASC speck formation was observed (Figure 7, H–J), strongly suggesting that inflammasome activation in astrocytes in vivo indeed requires AIM2.

## Discussion

Many studies show gene and protein expression of inflammasome components in the CNS in vivo, but a few have evaluated bona fide inflammasome activation. This study used reporter mice to detect activated inflammasomes in situ in a cell type–specific manner in the CNS during EAE. Using ASC-Citrine mice, we identified AIM2 inflammasome activation after disease peak mainly in astrocytes in the SC of EAE mice. Unexpectedly, we detected limited or no inflammasome activation in CNS-infiltrated myeloid cells or microglia.

Previous studies suggested inflammasome activation in CNS-resident cells during EAE and MS, but these studies evaluated inflammasome activation of microglia or astrocytes in tissue culture (9, 14–18). A few studies demonstrated inflammasome activation in the CNS during EAE in situ. One such study showed ASC specks in Iba-1^+^ cells, suggested to be microglia, in the hippocampus of EAE mice, but the investigation was not extended to astrocytes (12). Another study suggested caspase-8–mediated noncanonical NLRP3 inflammasome activation in microglia in the SC of EAE mice based on caspase-8-FLICA staining of tissue sections (31). It is possible that a small number of microglia may exhibit active inflammasomes, though our data showed that substantially more inflammasome activation occurred at 30 dpi in astrocytes.

We and other groups have shown that the NLRP3 inflammasome is detrimental in EAE. However, recent studies (14, 30) and our results demonstrate that AIM2 can play a protective role. Chou et al. demonstrated that AIM2 suppresses EAE by promoting Treg stability in an inflammasome-independent fashion (30). Similarly, the study by Ma et al. demonstrated a protective role of AIM2 through another inflammasome-independent mechanism targeting the DNA-PK-AKT3 pathway (14). Using *Aim2^fl/fl^ Gfap^Cre^* mice, Ma et al. evaluated the disease severity in Type-B EAE and found that astrocyte-specific *Aim2* depletion did not change the disease severity (14). Of note, the study did not evaluate the disease score beyond 18 dpi (around disease peak) (14). However, our data indicated numbers of ASC specks both in naive and 16 dpi EAE mice (around disease peak) were basal, while the most significant increase was observed on 30 dpi. Additionally, Ma et al. induced Type-B EAE in *Aim2^fl/fl^ Gfap^Cre^* mice (14) but we did not. Thus, intensity of EAE induction possibly affects the involvement of AIM2 in EAE too, as we demonstrated that increased adjuvant upon induction of EAE blunts the impact of AIM2 on disease (Figure 7F). A long-term evaluation of astrocyte-specific AIM2-knockout mice and elucidating an impact of EAE induction methods will merit further understanding of the protective role of the AIM2 inflammasomes in astrocytes.

Using the ASC-Citrine mouse model, 2 recent reports have identified ASC specks in the cerebellum during development (13) and in retinal astrocytes during ocular hypertension injury (32). Notably, 1 of the studies also showed the ASC specks in naive animals (13), mirroring our finding of ASC specks in naive SC (Figure 1, E and I). Further, the ASC specks in the study are AIM2 dependent, and the AIM2 inflammasome contributes to normal brain development (13). Therefore, the function of the AIM2 inflammasome in the CNS may be intrinsically different from that in peripheral myeloid cells, which are equipped to induce inflammation. For example, our study indicated that astrocytes express little IL-1β during EAE upon AIM2 inflammasome activation (Supplemental Figure 4, A, C, and F), although IL-1β release by astrocytes was identified in response to NLRP3 inflammasome activation in the tissue culture setting (Figure 6C and Supplemental Figure 5B). This suggests that inflammasome activation in astrocytes during EAE may have biological implications other than enhancing inflammation.

In this study, we observed ASC strings, which were unique to the CNS in vivo. However, some ex vivo studies have shown a similar ASC string-like structure. “ASC filaments” have been documented ex vivo with mutant ASC (33) or with ASC CARD domain blockade or deletion (34–36). An ASC isoform (ASC-c) also generates ASC filaments in human cells and appears to be expressed in mice, at least in the J774A.1 cell line (37). It is not known how astrocytes generate ASC strings, but several possibilities exist, such as potential astrocyte-specific expression of the ASC-c isoform or interaction of inflammasome components with astrocyte-specific molecules. For example, GFAP and vimentin bind together as part of the astrocyte intermediate filament network (38), and vimentin is known to interact with inflammasome components, such as caspase-1 (39). Thus, a possible physical association of GFAP to inflammasome components might explain the appearance of ASC strings in the highly ramified astrocyte.

We identified activated caspase-3 in astrocytes with ASC specks in the absence of cell death. Caspases, including caspase-3, possess critical functions outside of induction of cell death (40). Specifically, nonapoptotic caspase-3 activation is involved in the differentiation of numerous cell types, such as monocytes, neurons, and hematopoietic stem cells (40). Also, neurons, which derive from similar progenitors to astrocytes, rely on caspase-3 for dendrite and axon remodeling (41, 42) and synaptic plasticity (43). Caspase-3 activation in astrocytes is also associated with astrogliosis and not cell death (44–47). Specifically, caspase-3 activation in astrocytes is associated with cytoskeletal remodeling in a kainic acid–induced neurodegeneration model (45), reactive astrocytes following excitotoxic NMDA-induced neurodegeneration (47), and GFAP cleavage in an Alzheimer’s disease model (44). Indeed, our data suggested the involvement of AIM2 inflammasome in caspase-3 activation and GFAP cleavage. The nonapoptotic activation of caspase-3 in astrocytes is inducible in vitro and promotes expression of glutamate synthase and basic fibroblast growth factor mediated by astrogliosis (46). Our study now connects inflammasome activation in astrocytes to caspase-3 activation and so warrants further study into the role of caspase-3 in astrogliosis.

The AIM2 inflammasome is activated by double-stranded DNA (dsDNA) derived not only from microbes but also from endogenous sources as a sentinel of genotoxic stress and DNA damage. The AIM2 inflammasome protects from gastrointestinal toxicity and hematopoietic failure in total-body irradiation (48), which triggers DNA double-strand breaks (48) and nuclear membrane disruption (49). Indeed, the detection of dsDNA by AIM2 is required for normal neurodevelopment during periods of proliferative stress in neurons of the CNS (13). These studies demonstrated roles for AIM2 that are not abjectly inflammatory and serve to protect normal body function during episodes of genotoxic damage and during development, which positions the AIM2 inflammasome separately from the classical understanding of other inflammasomes, such as the NLRP3 inflammasome. Opposing outcomes of EAE severity in the absence of AIM2 versus ASC, as well as our results demonstrating a detrimental role of ASC in nonhematopoietic cells (Figure 2, A–C), are intriguing, but perhaps not surprising, as ASC is the common adaptor to other inflammasomes, including the NLRP3 inflammasome, which is pathogenic during EAE. Thus, the pathogenic impact of the NLRP3 inflammasome (and perhaps the Pyrin inflammasome; ref. 50) on EAE potentially supersedes the functions of the AIM2 inflammasome in an ASC-deficient animal.

In conclusion, our study demonstrates astrocyte AIM2 inflammasome activation without eliciting IL-1β–mediated inflammation in the late phase of EAE. This study expands our understanding of astrocytes in EAE and warrants further investigation of noninflammatory functions of the AIM2 inflammasome in astrocytes during neuroinflammation.

## Methods

### Animals.

We used mice of the C57BL/6 genetic background of both sexes aged 8–12 weeks old, unless otherwise noted. Because we did not identify sex differences in our experiments, both male and female were equally represented in our experiments. The ASC-Citrine mice were generated and gifted by Douglas Golenbock (University of Massachusetts Chan Medical School, Worcester, Massachusetts, USA). The *Pycard*^–/–^ and *Nlrp3*^–/–^ mice were initially obtained from Genentech. The following mice are from The Jackson Laboratory; *Cx3cr1*^CreERT2^ (stock 020940) *Gfap*^Cre^ (stock 012886), *Asc-Citrine^LSL^* (stock 030743), *Syn1*^Cre^ (stock 003966), and *Aim2*^–/–^ (stock 013144). *Gfap^Cre^* and *Syn1*^Cre^ mice were used as heterozygotes.

### EAE induction and scoring.

Mice were immunized with CFA/MOG emulsion in the lower back on day 0. The emulsion was prepared by mixing MOG_35-55_ peptide (United Biosystems, catalog U104628) and CFA (MilliporeSigma, catalog F5881) with additional *M. tuberculosis* (BD Difco, catalog 231141; 200 μg/mouse). The mice also received an intraperitoneal injection of Pertussis toxin (PTx) (200 ng/mouse) (List Biological Technologies, catalog 180) on days 0 and 2. Unless otherwise noted, we induced EAE with this method, as Type-A EAE (100 μg MOG_35-55_/mouse and 200 μg *M. tuberculosis*/mouse) (10). In some experiments, Type-B EAE (10) was induced with CFA/MOG injection on days 0 and 7 (100 μg MOG_35-55_/mouse, 400 μg *M. tuberculosis*/mouse) and PTx (200 ng/mouse) on days 0, 2, and 7. EAE was scored as previously described (2, 3, 10).

### Preparation of frozen tissue sections and staining with antibodies.

Animals were lethally anesthetized with 100 mg/kg of Nembutal administered intraperitoneally and transcardially perfused with PBS and subsequently 4% paraformaldehyde (PFA) (MilliporeSigma, catalog 158127). SCs and iLNs were harvested and fixed for 24 hours at 4°C in 4% PFA. All tissues were cryoprotected in 30% sucrose in water for an additional 24 hours before embedding and freezing in Tissue-Tek O.C.T. compound (Sakura, catalog 4583) on dry ice. Tissues were sectioned using a Cryostar NX50 (Thermo Fisher Scientific) at a thickness of 25 μm and rendered as floating sections. Sections were permeabilized with 0.25% Triton X-100 (Amresco, catalog 0694-1L) and blocked using 2% bovine serum albumin (GeminiBio, catalog 700-101P). After antibody staining, sections were mounted onto slides with ProLong Gold Antifade Mountant (Invitrogen, catalog P36931) or ProLong Gold Antifade Mountant with DAPI (Invitrogen, catalog P36930). TUNEL staining was also performed with 25 μm thick floating tissue sections with the CF 640R TUNEL Assay Apoptosis Detection Kit (Biotium, catalog 30074). Antibodies used for staining are indicated in Supplemental Table 1.

### Preparation of tissue slice culture and anti-ASC immunostaining.

Spleens were dissected out of mice, and live spleen slices were prepared with a vibratome (Precisionary Compresstome) using 4% low-melt agarose as described by manufacturer’s protocol. Slices in complete RPMI-1640 (Gibco) were kept as floating sections and treated with 100 ng/mL Ultrapure LPS (InvivoGen, catalog tlrl-3pelps) for 2 hours followed by 5 μM nigericin (MilliporeSigma, catalog N7143) for 1 hour. After fixation and permeabilization with ice-cold methanol for 15 minutes, slices were blocked with 2% BSA in PBS for 1 hour at room temperature. Immunofluorescence staining was performed with antibodies listed in Supplemental Table 1.

### Immunofluorescence microscopy and image analyses.

All slides were imaged on the Zeiss 710 Inverted Laser Scanning Confocal Microscope (Duke University Light Microscopy Core Facility) at full 25 μm depth as *Z*-stacks. For quantifications, 2 sections per animal were imaged, and a 2 × 2 grid tile scan was performed using the 20× objective centered on the VH of the SC, totaling 8 fields per mouse. Following quantification, these replicates were averaged to generate a single *n* for statistical analyses. Semiautomated quantification was conducted using the Imaris for Neuroscientists Cell Imaging Software ver. 9.3.0. (Bitplane) unless otherwise indicated. Briefly, the Surfaces tool was used to identify either ASC specks/strings or cells, and intensity thresholds of counterstain signals within the surfaces were used to quantify the desired characteristics. ASC specks and ASC strings counts in Figure 1 were manually enumerated using the LSM Browser software (Zeiss).

### BM chimeras.

Recipient mice (CD45.2; 6–8 weeks old) were lethally irradiated with 9 Gy (XRAD 320 x-ray irradiator) and adoptively transferred with 10^6^ CD45.1 donor BM cells. Mice were supported with water containing sulfamethoxazole and trimethoprim (Pharmaceutical Associates, Inc.) for 1 week following irradiation. At 6 weeks after adoptive transfer, the donor cell reconstitution was confirmed by differential expression of congenic markers in peripheral blood using the FACSCanto II (BD Biosciences).

### TAM pulse for selective microglial labeling.

This procedure was adapted from previous work (51). Briefly, *Cx3cr1^CreERT2^* mice (6–8 weeks old) received an intraperitoneal injection of 75 mg/kg TAM dissolved in corn oil. The second injection with the same formula was administered 2 days later. Mice were kept for 6 weeks after the last TAM administration for 6 weeks before EAE induction.

### Astrocyte isolation and RT-qPCR.

Astrocytes were isolated as previously described (52) with a few modifications. Briefly, mouse SC were minced into approximately 1 mm^2^ pieces and digested using the Papain Dissociation System (Worthington Biochemical Corporation, catalog LK003153). Isolated cells were negatively selected in tandem first with Myelin Removal Beads II (Miltenyi Biotec, catalog 130-096-731) and second with anti-CD11b MicroBeads (Miltenyi Biotec, catalog 130-049-601) to collect the flowthrough fraction. To enrich astrocytes, the flowthrough cells were first treated with FcBlock (Miltenyi Biotec, catalog 130-092-575), then positively selected using anti-ACSA-2 MicroBeads (Miltenyi Biotec, catalog 130-097-678). Total RNA was prepared using TRI Reagent (MilliporeSigma, catalog 93289) and reverse-transcribed using qScript cDNA Mix (Quantabio, catalog 950048) to obtain cDNA. RT-qPCR assays were performed with SYBR FAST qPCR Master Mix (Kapa Biosystems, catalog KK4602), using primers indicated in Supplemental Table 2. Expression levels of target genes relative to an internal control, *Actb*, were calculated using the *ΔΔCt* method (53).

### Cell culture and WB analysis.

BMDMs were generated by culturing total BM cells for 7 days in complete RPMI supplemented with 10 ng/mL recombinant mouse M-CSF (BioLegend, catalog 576406M-CSF). Preparation of primary cortical astrocytes was performed as previously described (54). Briefly, cortices were obtained from P4 pups, then enzymatically digested, and cells were cultured for 7 days in complete DMEM. Astrocytes were enriched by shaking of flasks to remove other cell types (54). One day before stimulation, BMDMs and cortical astrocytes were seeded into poly-l-lysine–coated, 12-well plates (1.5 × 10^6^ cells/well). Then, cells were pretreated with 100 ng/mL Ultrapure LPS (InvivoGen, catalog tlrl-3pelps) in serum-free Opti-MEM (Thermo Fisher Scientific, catalog 11058021) for 2 hours and stimulated with 5 μM nigericin (MilliporeSigma, catalog N7143) or poly(dA:dT) (InvivoGen, catalog tlrl-patn) for 4 hours to activate the NLRP3 and AIM2 inflammasomes, respectively. Poly(dA:dT) was used as a complex with Lipofectamine 2000 (Invitrogen, Thermo Fisher Scientific, catalog 11668019) as a concentration of 0.5 μg/mL. Culture supernatants and cell lysates (prepared in RIPA buffer, Thermo Fisher Scientific, catalog 89900) were harvested, and total protein concentrations were quantified using the BCA Protein Assay Kit (Thermo Fisher Scientific, catalog 23227). The same amount of total protein was used across all samples for SDS-PAGE gel separation. WB was performed with indicated antibodies (Supplemental Table 1), and the result was imaged using the GeneGnome Chemiluminescence System (Syngene) and the Genesys (Syngene) software. Band intensity was quantified using the ImageJ software. Lysate GAPDH band intensity was used to normalize both the lysate and supernatant band intensity.

### Statistics.

AUC values were used to statistically analyze EAE scoring data. The Mann-Whitney *U* test was used to compare between 2 groups, unless otherwise indicated. Prepeak and postpeak AUCs were defined by identifying the dpi at which disease score ceased to increase or began to decrease for both groups in a single experiment. All other analyses, where indicated in the figure legends, were performed using the Mann-Whitney *U* test, a 1-factor ANOVA, or a 2-factor ANOVA. Post hoc testing was performed only if ANOVA reached significance on interaction term (*P* < 0.1). Dunnett’s multiple comparisons and Holm-Šidák multiple comparisons were conducted post hoc where indicated in the figure legends. All statistical analyses were performed using the GraphPad Prism 8 software, and significance was defined if the differences between groups reached a *P* < 0.05 during testing.

### Study approval.

Mice for all experiments were housed in a specific pathogen–free environment. All animal experiments included in this study were approved by the Institutional Animal Care and Use Committee of Duke University.

## Author contributions

WEB, MED, and MLS designed research; WEB, MED, MI, TN, KN, NAL, and NA performed experiments; WEB, MED, and MLS analyzed data; WEB and MLS wrote the manuscript; and MED, MI, KN, NAL, NA, and EAM provided feedback on the manuscript.

## Supplementary Material

Supplemental data

Supplemental tables 1-2

## Version 1 04/22/2022

Electronic publication
