# Supplementary material for: The AIM2 inflammasome is activated in astrocytes during the late phase of EAE
Source: JCI Insight. 2022 Apr 22;7(8):e155563. doi: 10.1172/jci.insight.155563 (PMC9089781; doi:10.1172/jci.insight.155563)
Supplement: Supplemental data [file jciinsight-7-155563-s019.pdf]

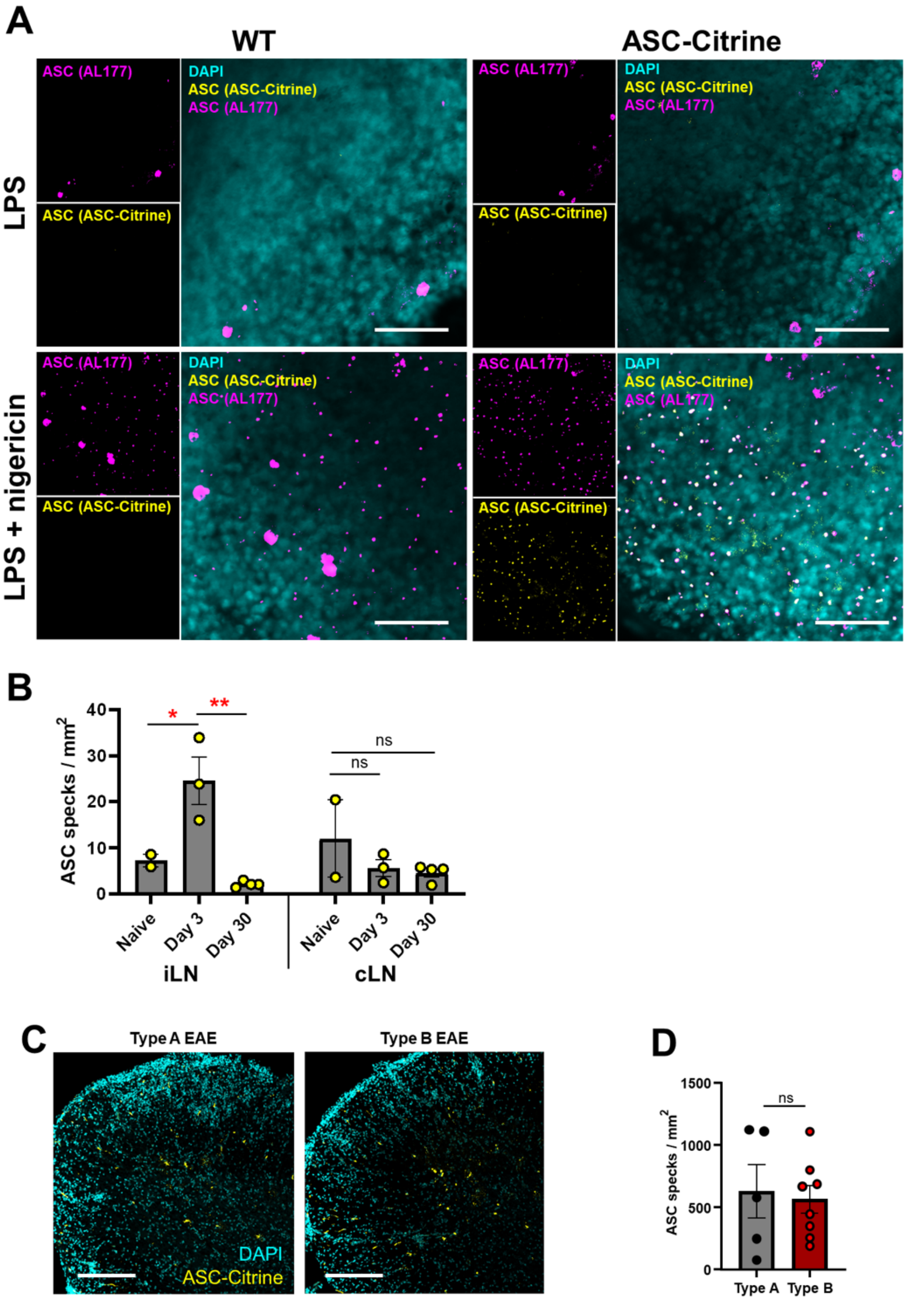

Supplemental Figure 1. Validation of ASC-Citrine system in tissue imaging. (A) Representative images of ASC speck formation detected with ASC-Citrine and ASC antibody signals. Live spleen tissue culture slices from naïve WT and ASC-Citrine mice were used with NLRP3 inflammasome stimulation. Scale bar is 50 µm. **(B)** Quantification of ASC specks in the iLNs and cLNs of ASC-Citrine mice during EAE. Each datapoint represents a value of an average value from two cross-sections of LNs (25 µm thickness) from one mouse. One-way ANOVA, p=0.0021 (iLN), p=0.3235 (cLN), with Dunnett's multiple comparisons test. **(C** and **D)** Comparison of ASC speck images and numbers in SC between Type-A and Type-B EAE. Representative images (C) and quantification (D) of ASC specks in the SC of ASC-Citrine mice at 30-dpi for Type A (n=5) and Type B (n=8) EAE. Each datapoint represents a value from one mouse. Mann-Whitney test was used *(D)*. Scale bar is 200 µm. (*B, D*) *ns*; not significant (p>0.05), *p<0.05, **p<0.01. Error bars denote mean ± SEM *(B, D)*.

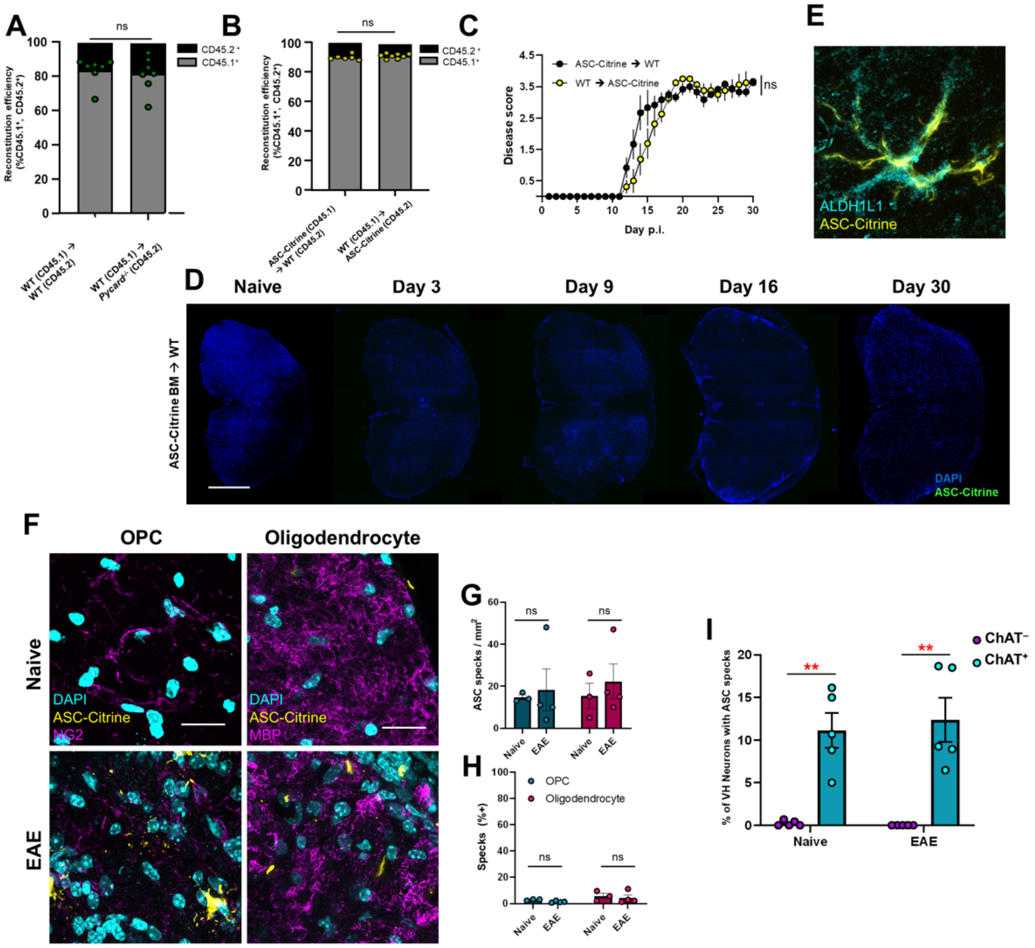

Supplemental Figure 2. Validation of Bone Marrow Chimeras. (A) BM chimera were created by transferring WT BM cells to irradiated WT or *Pycard*$^{-/-}$ recipients (n=7 for each group). Reconstitution efficiency of BM chimeras determined by flow cytometry, quantified as % of total CD45^{+} cells in peripheral blood for congenic markers of CD45.1 (donor) or CD45.2 (recipient). Each datapoint represents a value from one mouse. Mann-Whitney test used. **(B** and **C)** BM chimera were created by transferring ASC-Citrine BM cells irradiated WT recipients (ASC-Citrine → WT, *n*=6) and vice versa (WT → ASC-Citrine mice, *n*=8). Reconstitution efficiency (*B*) and EAE disease score (*C*) of indicated BM chimera. Each datapoint represents a value from one mouse *(B).* Each datapoint denotes mean EAE score per group, and Mann-Whitney test of total AUC was used for statistical evaluation *(C)*. **(D)** Representative images of SC from WT recipients reconstituted with ASC-Citrine BM cells at indicated time points during EAE. No apparent ASC specks were observed. Scale bar is 500 µm. **(E)** Representative image of ALDH1L1 counterstaining of astrocytes in ASC-Citrine mice at 30-dpi EAE. Scale bar is 10 µm. **(F)** Representative images of ASC specks and strings counter-stained with antibodies against NG2 (for OPCs) and MBP (for mature oligodendrocytes) in SC from naïve versus 30-dpi EAE ASC-Citrine mice. Scale bar is 20 µm. **(G)** Quantification of ASC specks in OPCs and mature oligodendrocytes of SC from naïve versus 30-dpi EAE ASC-Citrine mice. Each datapoint represents a value from one mouse. Two-way repeated measures (RM) ANOVA was used (main effect of cell type: $^{ns}p<0.7807$). **(H)** Percentages of ASC specks detected in OPC or mature oligodendrocytes out of total ASC specks per section. L5 spinal cords at 30-dpi EAE were used for the analysis. **(I)** Percentage of ChAT^{+} and ChAT^{-} VH neurons containing ASC specks in SC from naïve vs. 30-dpi EAE ASC-Citrine mice. Each datapoint represents a value from one mouse *(n=5).* Two-way RM ANOVA was used (main effect of cell type: *p<0.001*) with Sidak's multiple comparisons test post hoc. *ns*; not significant (*p>0.05*), ***p<0.01*. Error bars denote mean ± SEM *(A, B, G-I).*

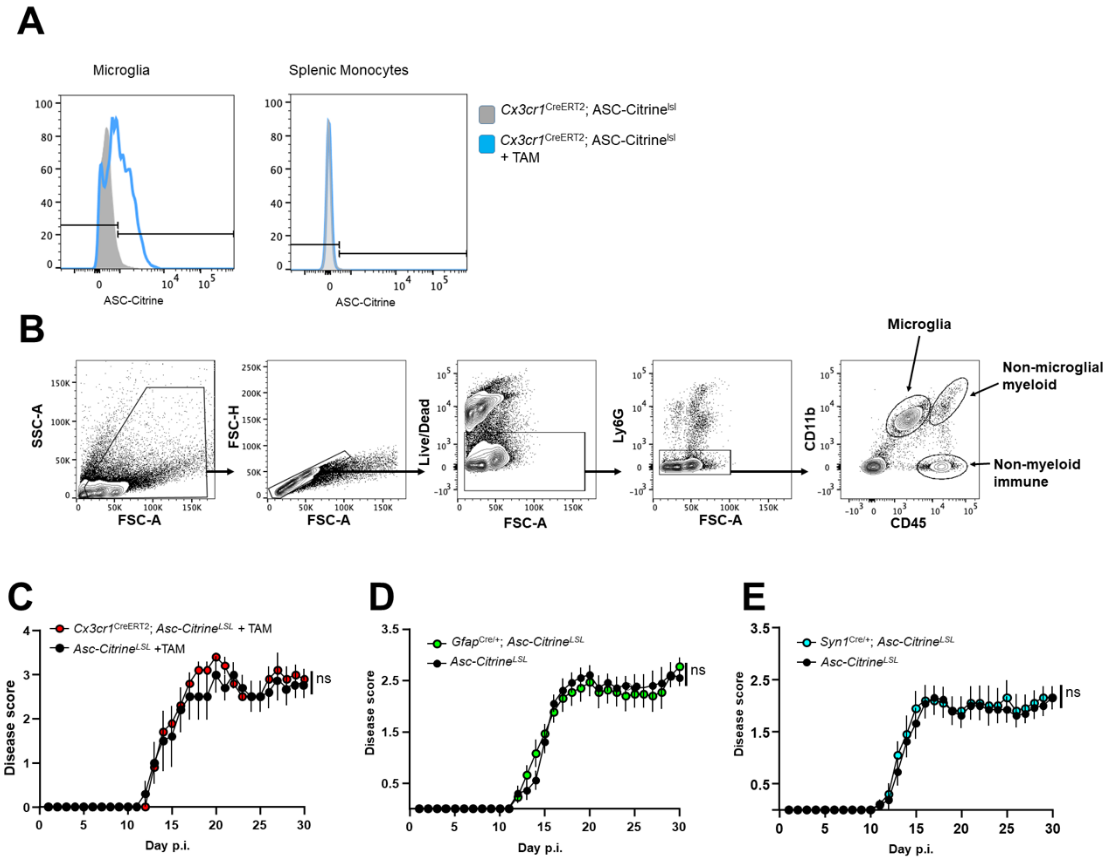

Supplemental Figure 3. Validation of EAE mice with cell type-specific ASC-Citrine expression. (A) Flow cytometry histograms showing tamoxifen-mediated expression of ASC-citrine reporter expression in microglia and splenic monocytes from *Cx3cr1*^{CreERT2}*;Asc-Citrine*^{LSL} mice with or without tamoxifen (TAM) treatment. **(B)** Flow cytometry gating strategy for identifying microglia. **(C)** EAE disease score of *Cx3cr1*^{CreERT2}*;Asc-Citrine*^{LSL} (*n=5*) vs. *Cx3cr1*^{CreERT2}*;Asc-Citrine*^{LSL} (*n=5)* mice. Both groups were treated with TAM. **(D** and **E)** EAE disease score of *Asc-Citrine*^{LSL} (*n=10*) vs. *Gfap*^{Cre}*;Asc-Citrine*^{LSL} (*n=13*) (*D*) and *Asc-Citrine*^{LSL} (*n=13*) vs. *Syn1*^{Cre}*;Asc-Citrine*^{LSL} (*n=10*) (E). Mann-Whitney test of total AUC of disease score was used for statistical analysis (*C, D, E*). *ns*; not significant (p>0.05). Each datapoint denotes mean EAE score per group with an error bar of mean ± SEM *(C-E*).

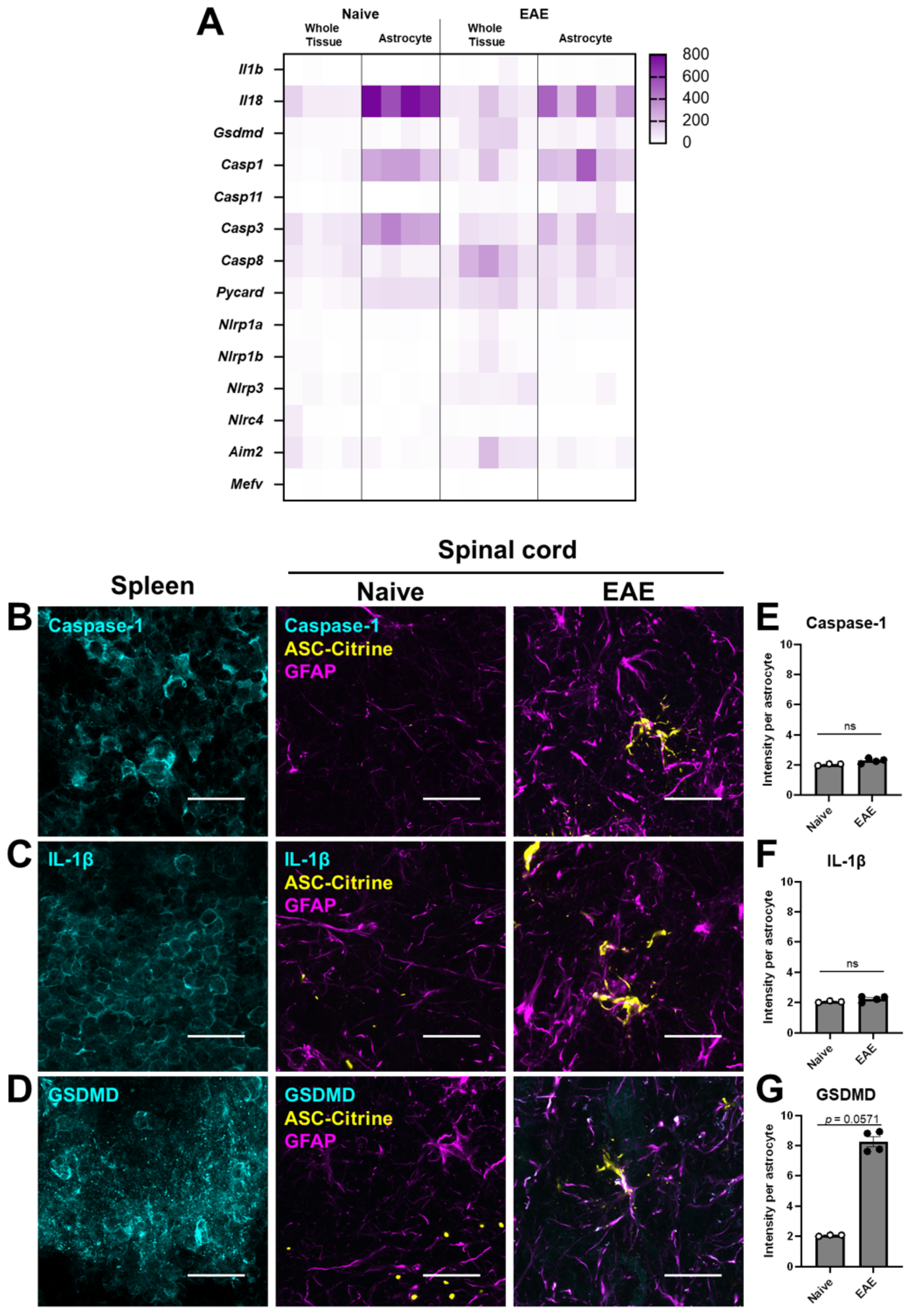

Supplemental Figure 4. Expression of inflammasome components and cell death markers in astrocytes during EAE. (A) Gene-set enrichment analysis of inflammasome-associated genes in bulk SC lysates and astrocytes (with astrocyte-specific Ribotag-HA enriched RNA) in naïve and 30-dpi EAE mice. Data represented as raw transcript counts derived from publicly available data (GEO Accession #: GSE100329). **(B-G)** Representative images (*B-D*) and quantification (*E-F*) of caspase-1 (*B*, *E*), IL-1β (*C*, *F*), and GSDMD (*D*, *G*) expression in spleen and SC astrocytes from naïve versus 30-dpi EAE ASC-Citrine mice. Scale bar is 20 µm. Each datapoint represents a value from one mouse. Individual astrocytes were identified using the Imaris software and the mean intensity per cell was quantified for caspase-1 (*E*), IL-1β (*F*) and GSDMD (*G*). Mann-Whitney test was used. (*E-G*) *ns*; not significant (p>0.05). Error bars denote mean ± SEM *(E-G)*.

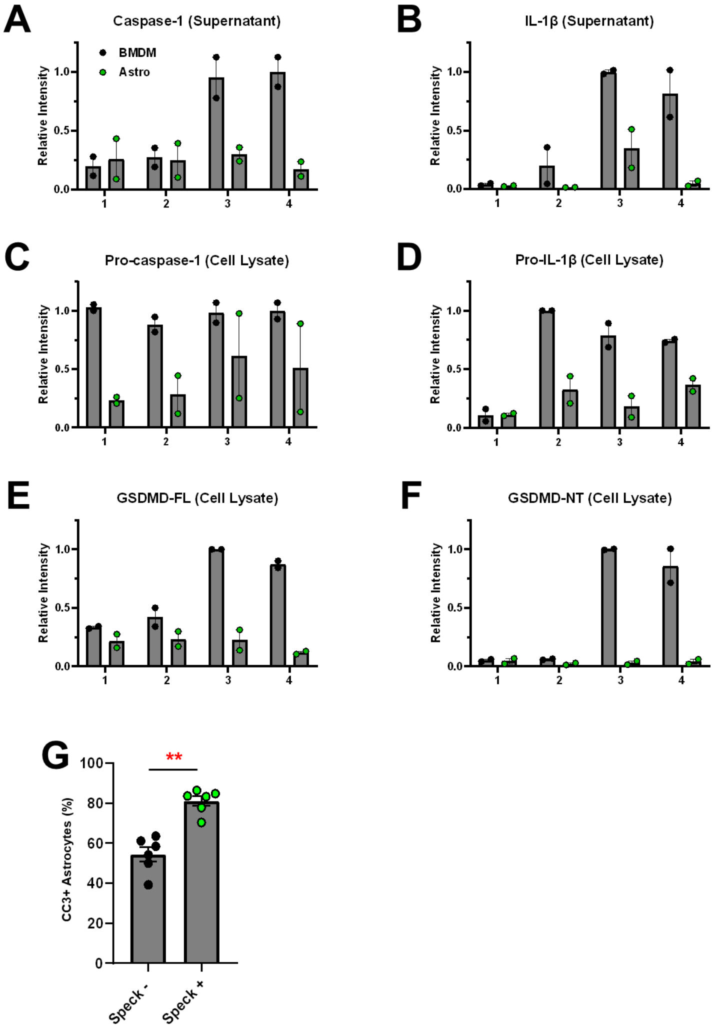

Supplemental Figure 5. Expression of inflammasome components in primary cortical astrocyte cell line (A and **B)** WB quantitative evaluation of culture supernatant samples of mature caspase-1 (*A*) and IL-1β (*B*). **(C-F)** WB quantitative evaluation of cell lysate samples of pro-caspase-1 (*C*), pro-IL-1β *(D),* GSDMD-FL (*E),* and GSDMD-NT (*F*). In (*A-F*), Cells in group 1 were unstimulated. Cells in group 2 were treated with Ultrapure LPS alone. Cells in groups 3 and 4 were pre-treated with Ultrapure LPS, and were further stimulated with nigericin and poly(dA:dT)/liposome to activate the NLRP3 and AIM2 inflammasomes, respectively. **(G)** Quantification of active caspase-3 (CC3) in spinal cord astrocytes comparing astrocytes with or without ASC specks in $Gfap^{Cre}$;$Asc\text{-}Citrine^{LSL}$ (*n*=6) mice at 30-dpi EAE. Each datapoint represents a value from one mouse. Individual astrocytes were identified using the Imaris software and were quantified by CC3 puncta staining. Mann-Whitney test was used. **$p<0.01$. Error bars denote mean ± SEM.

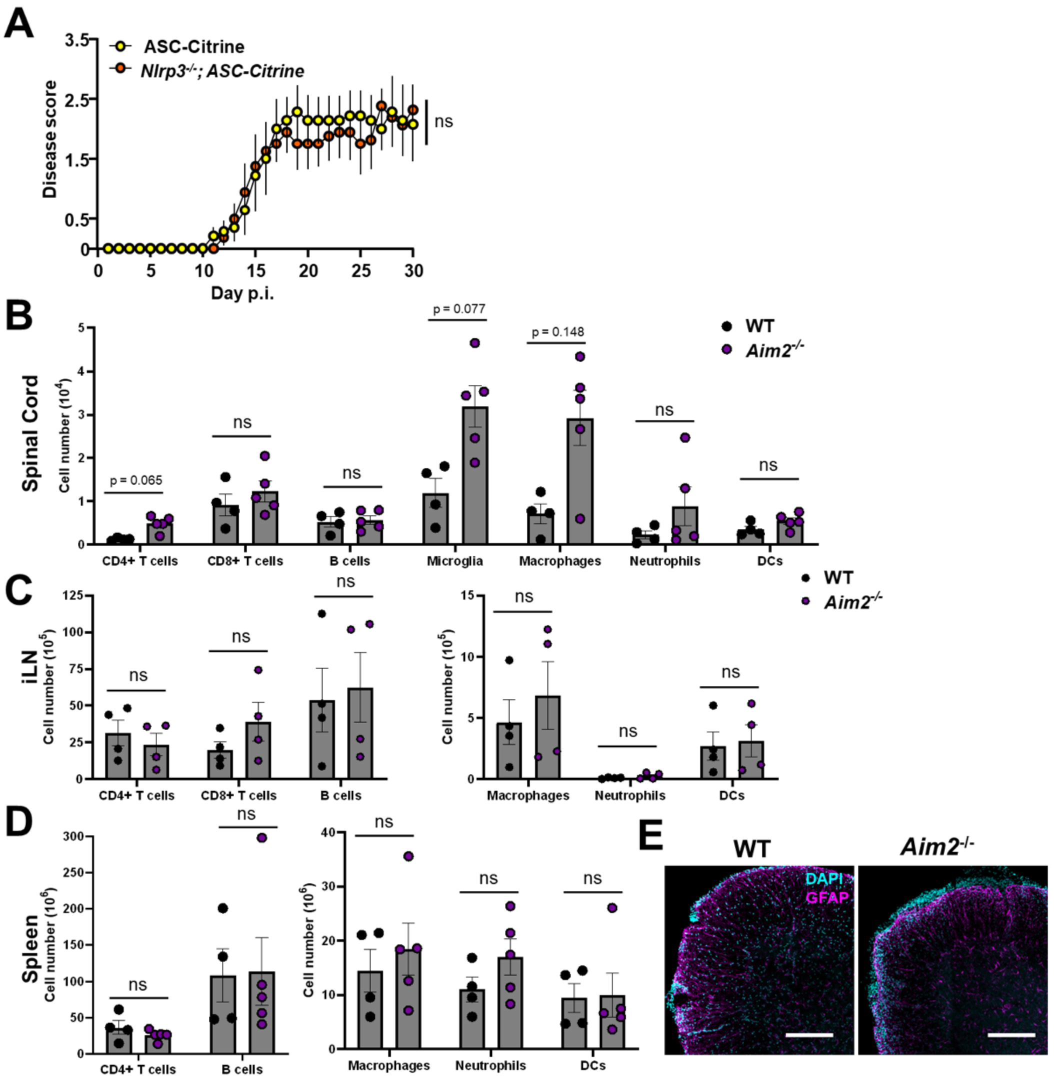

Fig. S6. Validation of EAE phenotype of *Nlrp3*$^{-/-}$;ASC-Citrine mice and immune phenotype of *Aim2*$^{-/-}$ mice with EAE. (A) EAE disease score of ASC-Citrine (*n=7*) vs. *Nlrp3*$^{-/-}$;ASC-Citrine (*n=8*) mice with Type B-EAE. Mann-Whitney test of total AUC for disease score was used. Each datapoint denotes mean EAE score per group with an error bar of mean ± SEM. **(B–D)** Leukocyte counts in SC (*B*), iLN (*C*) and spleen (*D*) at 16-dpi EAE in WT vs. *Aim2*$^{-/-}$ mice induced with Type-A EAE. Each datapoint represents a value from one mouse Error bars denote mean ± SEM. Two-way RM ANOVA was used with Sidak's multiple comparisons test post hoc. **(E)** Representative image of GFAP staining in SC from WT versus *Aim2*$^{-/-}$ mice at 30-dpi of EAE. Scale bar is 200 μm. *ns*; not significant (p>0.05) (*A-D*).
