# Supplementary material for: The AIM2 inflammasome is activated in astrocytes during the late phase of EAE
Source: JCI Insight. 2022 Apr 22;7(8):e155563. doi: 10.1172/jci.insight.155563 (PMC9089781; doi:10.1172/jci.insight.155563)
Supplement: Supplemental tables 1-2 [file jciinsight-7-155563-s020.pdf]

Table S1. Reagent information for antibody-based techniques

Target	Cat #	Species	Clone	Supplier	Dilution	Technique
ASC	AG-25B-0006-C100	Rabbit	AL177, polyclonal	Adipogen	1:500	IF Imaging
Tmem119	ab209064	Rabbit	28-3	Abcam	1:500	IF Imaging
GFAP	13-0300	Rat	2.2B10	Invitrogen	1:500	IF Imaging, Western Blotting
ALDHL1	ab87117	Rabbit	polyclonal	Abcam	1:500	IF Imaging
NeuN	ab104225	Rabbit	polyclonal	Abcam	1:500	IF Imaging
ChAT	AB144P	Goat	polyclonal	EMD Millipore	1:100	IF Imaging
NG2	ab275024	Rabbit	EPR23976-145	Abcam	1:500	IF Imaging
MBP	ab218011	Rabbit	EPR21188	Abcam	1:500	IF Imaging
Iba1	NB100-1028	Goat	polyclonal	Novus Biologicals	1:500	IF Imaging
C3d	AF2655	Goat	polyclonal	R&D Systems	1:500	IF Imaging
Cleaved caspase-3	9664S	Rabbit	5AIE	Cell Signaling Technologies	1:500	IF Imaging
Caspase-1	N/A	Rat	4b4	Genentech	1:500	IF Imaging, Western Blotting
Gasdermin D	PA5-1155330	Rabbit	polyclonal	ThermoFisher Scientific	1:500	IF Imaging
Gasdermin D	ab209845	Rabbit	EPR19828	Abcam	1:500	Western Blotting
IL-1β	AF-401-NA	Goat	polyclonal	R&D Systems	1:1000	IF Imaging, Western Blotting
IL-18	210-401-323	Rabbit	polyclonal	Rockland	1:1000	Western Blotting
CD45.1	110708	Mouse	A20	Biolegend	1:200	Flow Cytometry
CD45.2	109831	Mouse	104	Biolegend	1:200	Flow Cytometry

Table S2. Sequences of primers used for RT-qPCR assays

Gene	Direction	Sequence
Actb	Forward	TGT TAC CAA CTG GGA CGA CA
	Reverse	CTG GGT CAT CTT TTC ACG GT
Casp1	Forward	GAA GGC CCA TAT AGA GAA AGA TTT TAT TG
	Reverse	GAC AGG ATG TCT CCA AGA CAC ATT
Gsdmd	Forward	GCG ATC TCA TTC CGG TGG ACA G
	Reverse	TTC CCA TCG ACG ACA TCA GAG AC
Il1b	Forward	CGC AGC AGC ACA TCA ACA AGA GC
	Reverse	TGT CCT CAT CCT GGA AGG TCC ACG
Il18	Forward	CAG GCC TGA CAT CTT CTG CAA
	Reverse	CTG ACA TGG CAG CCA TTG T
